# Does genetic testing impact stigma in autism: A scoping review

**DOI:** 10.1007/s12687-025-00854-8

**Published:** 2026-01-28

**Authors:** Ciara J. Molloy, Adam Miles, Claire Christoff, Jessie Cunningham, Jamie Reilly, Jacob Vorstman, Jehannine Austin, Lisa D. Hawke, Louise Gallagher

**Affiliations:** 1 Department of Psychiatry, School of Medicine, Trinity College Dublin, Dublin, Ireland; 2 The Peter Gilgan Centre for Research and Learning, SickKids Research Institute, Toronto, ON Canada; 3 Health Sciences Library, The Hospital for Sick Children (SickKids), Toronto, ON Canada; 4 Technology Services Directorate College of Science and Engineering, University of Galway, Galway, Ireland; 5 University of British Columbia, Vancouver, BC Canada; 6 The Centre for Addiction and Mental Health, Toronto, ON Canada; 7 Department of Psychiatry, Temerty Faculty of Medicine, University of Toronto, Toronto, ON Canada

**Keywords:** Autism, Genetic testing, Self-stigma, Public stigma, Affiliate stigma

## Abstract

Public stigma is a society’s negative attitude toward a person or groups of people, self-stigma relates to the internalisation of these negative attitudes, and affiliate stigma is experienced by those associated with a stigmatised group. Both autism and genetic testing results can be stigmatised. This scoping review explored relationships between genetic testing and stigma in autism, to understand any perceived positive or negative effects. Embase Classic, and APA PsycINFO via Ovid databases were searched on May 30th, 2023, and updated January 9th, 2025. Included articles related to experiences of or attitudes toward genetic testing or genetic counselling, and involved autistic people, people with autism-related genetic conditions, or their parents, and explored stigma and stigma-related factors. Twenty-five articles were included: three included autistic adults and twenty-two included parents of autistic people or those with autism-related genetic conditions. Stigma-related factors included both autistic people’s and parent concerns surrounding social and insurance discrimination, parental experiences of isolation and ignorance in relation to the healthcare system, and perceptions of labelling. Findings were mixed regarding self-blame, worry and stress among autistic adults. Some parents reported an increase in worry and stress relating to discrimination, while others reported relief related to genetic test results and enhanced biological understanding. This review highlights the lack of focus on perspectives of autistic adolescents and individuals with autism-related genetic conditions, regarding genetic testing impacts on stigma. Future research should address these gaps to inform more supportive clinical and societal practices.

**Supplementary Information:**

The online version contains supplementary material available at 10.1007/s12687-025-00854-8.

## Introduction

Autism spectrum condition (henceforth autism) is a complex neurodevelopmental condition, associated with significant phenotypic variability and biological heterogeneity. It is well established that genetic factors strongly influence the development of autism in tandem with less well-elucidated environmental factors (Ronald and Hoekstra 2011; Modabbernia et al. 2017; Hallmayer et al. 2011; Deng et al. 2015; Leblond et al. 2024). The American Academy of Pediatrics (AAP) recommends offering genetic testing to all families as part of the evaluation of underlying etiology (Hyman et al. 2020); the National Institute for Health and Care Excellence (UK) recommends genetic testing only if an underlying genetic condition is suspected (www.nice.org.uk/guidance/cg142). The American College of Medical Genetics and Genomics 2013 guidelines recommended offering clinical genetic testing and evaluation for every autistic individual (Schaefer and Mendelsohn 2013), however, the most recent recommendations focused on congenital anomalies and intellectual disability, and evaluation of autism without an intellectual disability or congenital anomaly remains an area of ongoing research (Manickam et al. 2021). Despite the integration of genetic testing into standard autism care in many countries, the wider impact of genetic testing on autistic individuals themselves and their families remains insufficiently understood (Byres et al. 2023; Diedericks et al. 2024). This includes the influence on psychosocial dimensions, such as societal attitudes and associated mental health effects (Thapar and Rutter 2021).

Stigma has been shown to strongly impact quality of life and mental health (Turnock et al. 2022). Public stigma can be defined as a society’s negative attitudes toward a person or group, whereas self-stigma is the internalised prejudice experienced by those who are targets of public stigma (Corrigan 2011). Affiliate stigma is directed towards and experienced by those who are associated with a stigmatized individual, such as family members. Evidence suggests that each of these types of stigma can impact mental health and well-being (Perry et al. 2022; Werner and Shulman 2013; Song et al. 2018).

Many factors contribute to stigma, such as labelling, stereotyping, ignorance, social isolation, prejudice and discrimination (Link and Phelan 2001). These factors can help to understand the context in which stigma can arise. For example, labelling can result in social separation when an individual is described as “different”, and stereotyping can result from a labelled “difference” being associated with a negative trait. Both discrimination and prejudice can arise through rejection experienced by individuals who are labelled. Stigma and these contributing factors can arise based on societal attitudes or cultural beliefs, which can significantly impact on people’s lives. Notably, while labelling by others can be experienced as stigmatising, when an individual or group claims a label for themselves, it can be empowering as it helps people to define who they are (Mogensen and Mason 2015).

Autistic people report that they view autism to be value neutral, whilst feeling that society perceives autism as a bad trait to have (Botha et al. 2022). This has led to a dilemma for some individuals regarding decisions to disclose their diagnosis or not (Botha et al. 2022). Greater levels of social camouflaging, which relates to masking of autistic traits to align with “social norms”, have also been linked to experiences of stigma in autistic individuals (Perry et al. 2022). The societal impact on autism-related stigma has influenced the identity-first language shift, using the term ‘autistic person’ instead of ‘person with autism’ to reshape societal views of autism and reduce deficit-based language (Kenny et al. 2015; Botha et al. 2022; Taboas et al. 2023). Many factors continue to contribute to autism-related stigma in different contexts, including public and professional knowledge, as well as differences in cultural beliefs surrounding neurodevelopmental conditions (Turnock et al. 2022; Kim et al. 2021). Although there has been improvement in societal attitudes, a recent report on neurodivergence from the Embracing Complexity Network (https://embracingcomplexity.org.uk/) cited that the top two research priorities, based on input from neurodivergent people, their families or carers, researchers, educational professionals and health professionals, are to reduce discrimination and overcome stigma toward neurodivergent people.

In psychiatric conditions, evidence suggests that viewing psychiatric conditions as genetic reduces stigma toward the individual but increases stigma toward their families (Phelan 2005; Yao et al. 2020). However, stigma is a multifaceted construct, and so attributing psychiatric conditions to genetics may decrease some aspects of stigma, including blame, while also increasing other aspects, including pity or desire for social distance (Haslam and Kvaale 2015; Yao et al. 2020). In the context of psychiatric genetic testing, stigma could also stem from the feelings of loss of control over the condition due to over-emphasising the genetic and biological factors (Kotzé and Zwide 2022). Provision of clear information during genetic counselling to reduce any misinformation has been emphasised to reduce a person’s self-stigma and to gain a stronger sense of agency over their condition (Huynh et al. 2023).

Insights from neurodevelopmental research show evidence that stigma increases vulnerability to poorer mental health outcomes and well-being for autistic individuals (Han et al. 2022). Caregivers of autistic individuals also report stigma-related increases in psychological distress and well-being (Papadopoulos et al. 2019) However, less is known about how the introduction of genetic testing has impacted stigma in autism, in either a positive or negative way, and any subsequent influence on mental health and well-being.

Given the increase in clinical genetic testing as part of autism assessment, which may lead to further testing in family members, there is a need to understand the impact of genetic testing on both autistic people and their families. While there have been extensive advancements in clinical genetic testing for autism, the uncertainty about the outcomes of the clinical genetic diagnosis, and low predictive validity in many cases remains a concern (Gandal et al. 2016; Ní Ghrálaigh et al. 2023; Morris et al. 2022). Stigma and discrimination may therefore arise from misinterpretation or limited understanding of genetic test results, highlighting the need to fully understand whether this is a concern for autistic people and their family members, and if so, how to mitigate the possibility of further stigmatisation (Spriggs et al. 2008; Biesecker and Peay 2003).

The objective of this scoping review was to search the existing research literature on the perspectives and experiences of genetic testing among autistic individuals, individuals with autism-related conditions and their family members, to understand the perceived effect on stigma, and potentially on mental health outcomes. Our main aim was to understand any positive or negative effect of genetic testing or counseling on stigma in autism. Our secondary aim was to understand if there was any subsequent positive or negative impact on mental health for autistic people, people with an autism-related genetic diagnosis, or their parents or family members.

## Methods

### PICO framework to define scoping review research question

The four key components of the PICO framework (participants, intervention, comparisons and outcomes) were used to define the scoping review research question. The PICO framework was implemented as the aim was to explore the diverse research conducted on the topic of stigma, autism and genetic testing. Participants included in the reviewed research articles were autistic people, people with autism-related genetic conditions or variants (see supplementary Table 1 for the full list) or their parents or caregivers; the intervention of interest was genetic testing; the comparisons were perspectives and experiences of genetic testing and counselling in autistic people, people with autism-related genetic conditions, and their parents or caregivers; the primary outcome was the impact of genetic testing or counselling on stigma, and the secondary outcome was any subsequent impact on mental health and well-being.

### Protocol and registration

The scoping review protocol was drafted using the Preferred Reporting Items for Systematic Reviews and Meta-analysis Protocols extension for Scoping Reviews (PRISMA-ScR). The final protocol was registered prospectively with the Open Science Framework on July 19th 2023 (10.17605/OSF.IO/DBKXE).

### Data sources and search strategy

The search strategy was developed by a professional medical librarian (JC) trained in knowledge synthesis at the Hospital for Sick Children’s Library. Searches were executed in Embase Classic, and APA PsycINFO via Ovid databases on May 30, 2023. The search strategy consisted of both controlled vocabulary, such as the National Library of Medicine’s Medical Subject Headings (MeSH) and Emtree Subject Headings (Embase), APA PsycINFO subject headings, and keywords. No date limits were applied. Studies were limited to English language publications. The reference list of the final set of included articles were also screened to identify and include any other relevant cited articles. The search was updated on January 9, 2025, using the same parameters (see Supplementary Materials for the detailed search strategy).

### Inclusion & exclusion criteria

Inclusion criteria were any articles related to the topics of either genetic testing and/or genetic counselling, participants who were either autistic people or people with autism-related genetic conditions (see supplementary materials for defined list of neurodevelopmental copy number variants (CNV) and monogenic conditions that were selected and included based on literature) or the parent of an autistic individual or carrier of related genetic condition, and articles with qualitative or quantitative results regarding experiences or attitudes towards genetic testing or genetic counselling in autism or related genetic conditions. Exclusion criteria were any reviews, meta-analysis, case studies, conference abstracts, and articles with no available full text or no available full text in English.

### Screening

Screening of articles was completed by three researchers (AM, CC, CJM) in parallel and independently. Rayyan software was used to track the screening process (Ouzzani et al. 2016). First articles were screened for duplicates, then for inclusion criteria by title and abstract, and finally by full text. To ensure a high level of consistency between researchers, titles and abstracts for the first 100 articles were screened and discussed between researchers for any discrepancies. This stage was also used to refine any criteria necessary to capture articles to be included. Reviewers agreed on 96% of the screening decisions for the first 100 articles. At the end of both title and abstract and full text screening and filtering stages, any discrepancies were first resolved through discussion between researchers, and any unresolved discrepancies were reviewed by the third researcher (CJM) for a final decision on inclusion when necessary. A total of 19 articles were discussed and inclusion decision resolved at title and abstract screening, and 8 articles at full text stage.

### Data extraction

Participant sample characteristics (including participant group, sample size, age, and gender) were extracted, as well as article content including the topic (genetic testing or counselling), whether results included participant perspectives or experiences collected using questionnaires, surveys and/or interviews, and qualitative or quantitative data related to genetic testing and/or counselling, stigma, and mental health impacts. In relation to stigma, we extracted information about type of stigma referenced in any of the articles (public, self, or affiliate), and any mention of the following stigma-related factors: isolation, prejudice, discrimination, labelling, stereotyping, rejection, ignorance, status loss, self-esteem, marginalisation (Link and Phelan 2001). These factors were defined prior to screening of articles. Societal level discriminatory practices, such as insurance or healthcare discrimination, were classified under public stigma for the purpose of data extraction and reporting of results. For mental health, we extracted data relating to mental health impacts, such as stress, distress, worry or anxiety on the child or parent specifically related to genetic testing and stigma.

Other article information extracted included the title, authors, journal, PMID, year of publication, institution and country in which the research study was conducted, which was used to identify any overlaps in studies data and/or samples.

## Results

### Final articles included following screening and filtering

**Fig. 1:**
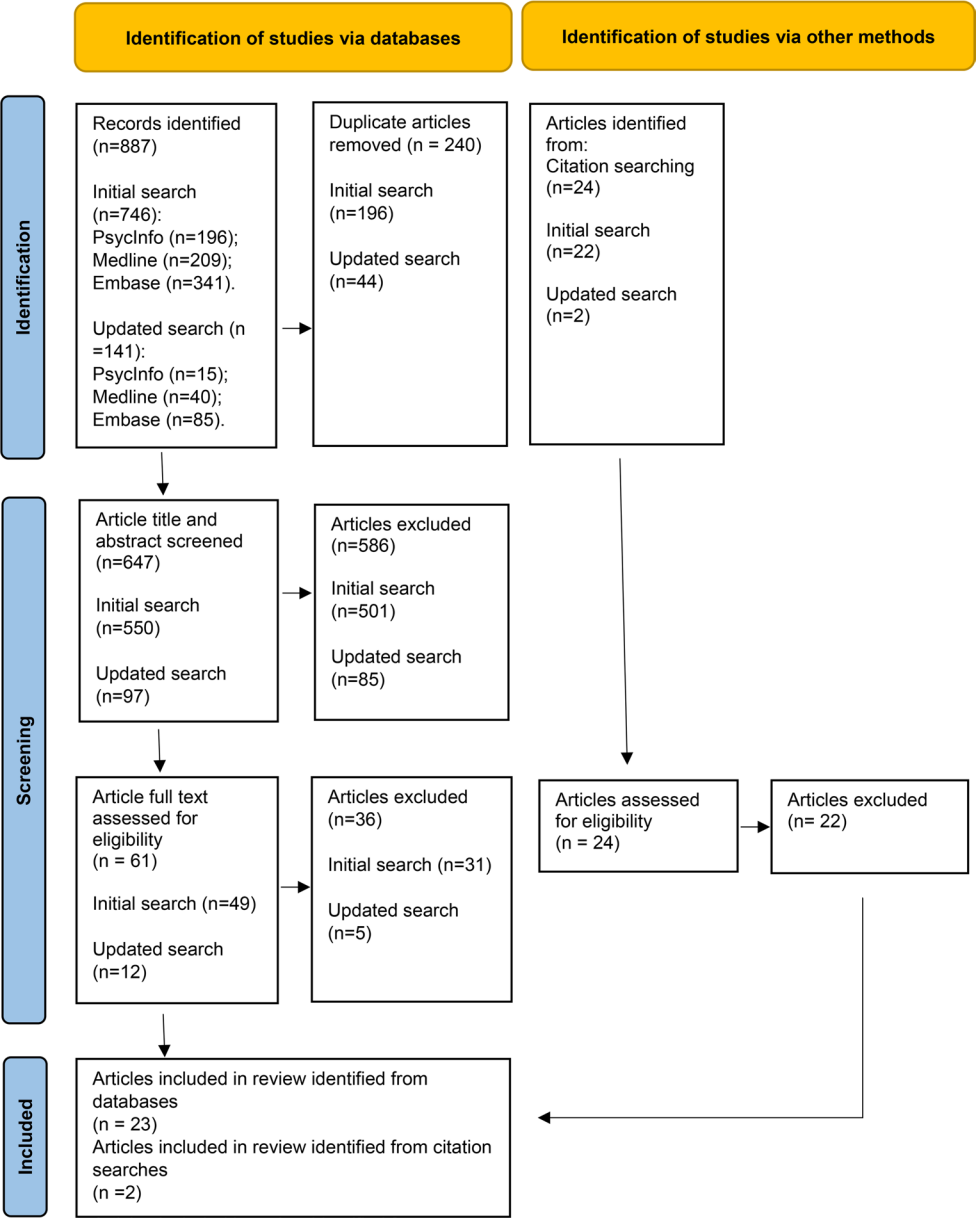
PRISMA Flow Diagram for Scoping Review

A total of 887 articles were returned from Library searches, following their search criteria (see supplementary materials for search strategy). Forty-four articles regarding experiences or attitudes towards genetic testing and/or genetic counselling in autism or related genetic conditions were identified based on search terms and inclusion and exclusion criteria defined for the scoping review. Twenty-three of the forty-four articles included results relating to stigma, or stigma-related factors defined for the scoping review data screening and data extraction (Link and Phelan 2001). We also identified and included two relevant articles cited in the final set of included articles relating to stigma, resulting in 25 articles included overall (Fig. 1).

### Study and participant characteristics

Three articles included autistic participants, and participants from 21 articles included parents or family members (Table 1). Eighteen of those included parents of autistic people and three included parents of people with autism-related genetic conditions. One parent study focused specifically on perspectives of parents of non-speaking autistic people (Asbury et al. 2024). Two studies reported on the same cohort (Johannessen et al. 2017; Johannessen et al. 2022). All the article topics were focused on genetic testing. Two studies were related to genetic services including access to genetic counsellor or geneticist, however the stigma results reported were related to genetic testing and not the impact of receiving genetic counselling. Many of the articles discussed education and the provision of information both before and after commencing clinical genetic testing. The majority of the studies were conducted in the United States of America (USA) (USA = 12; Canada = 5; Norway = 2 based on same cohort; Taiwan = 1, The Netherlands = 1; Ireland = 1; England = 1; South Africa = 1; Worldwide = 1). The articles reported on findings from either a clinical or a research setting.

**Table 1:** Summary of participant characteristics, where reported, across studies included in the scoping review

Participant Characteristics	
Sample Size (n, range)	9–1455
Autistic Adult Gender (f, range %)	54–64%
Parent Gender (f, range %)	57–95%
Child Gender (f, range %)	17–41%
Autistic Adult Age (range)	18–67
Parent Age (range)	22–90
Child Age (range)	0–58

### Public stigma

#### Autistic people’s perspectives and experiences

There were mixed findings in autistic people’s perspectives relating to the impact of genetic testing on public stigma and societal acceptance of autism. The majority of autistic adult participants in a larger sample study (*n* = 461), were not interested in genetic testing despite believing that genetics contributes to autism (Byres et al. 2023). Over half (61%) of participants did not believe that genetic testing would enhance societal acceptance of autism. Among autistic individuals who had not undergone genetic testing, just over half (53%) reported that they did not expect increased acceptance from their families. Notably, a higher proportion (71%) of those who had received genetic testing indicated that it did not lead to greater familial acceptance (Byres et al. 2023). Similarly, many autistic participants in another study with a large sample size (*n* = 173) expressed concerns that genetic testing would pathologise autism and exacerbate stigma (Gallion et al. 2025). In a smaller qualitative, semi-structured interview-based study, some participants believed that genetic testing would reduce stigma and increase acceptance through validation of a diagnosis, increased understanding and enhanced public awareness (Klitzman et al. 2024).

#### Parent perspectives and experiences

Public stigma was referenced by parent participants in seven articles (Table 2), with mixed findings reported. In a study of 97 parents, the majority of participants (85%) agreed that genetic testing could reduce public stigma by increasing knowledge of the underlying biological mechanisms, which could help to perceive autism as a biological condition rather than a behavioural condition (Yusuf et al. 2020). In another study comparing parent and scientist perspectives (*n* = 502) on causes of autism, stigma and genetic research impacts, the majority (70%) of parent participants thought that genetic testing would not influence the stigma experienced in autism, while a quarter (26%) thought that it might reduce it (Fischbach et al. 2016). In another study of parents (*n* = 45), about half (47%) reported that a motivator for testing was the potential to enhance the understanding of underlying causes of autism (Xu et al. 2016). In a smaller qualitative study sample (*n* = 9), parents indicated that the value of a genetic result was related to increasing awareness and recognition of autism, which could in turn reduce public judgement of both parents and their child (Trottier et al. 2013). Finally, in a sample of 155 parents, a subset (10%) who expressed disinterest in genetic testing, cited concerns that clinical genetic services would stigmatise autistic children (Vande Wydeven et al. 2012).

**Table 2:** Summary of research articles included in the scoping review that describe stigma and/or stigma-related factors in the context of autism and genetic testing

Author	Study Focus*	Data Type	Cohort	Child/Adult Characteristics	Parent Characteristics	Stigma & Related Factors Results
(Asbury et al. 2024)	Perspective	Qualitative	Autism (parent)	*n* = 21 Gender (f) = 33.3% Age: range = 4- 11yrs	*n* = 20 Gender (f) = 95%	Prejudice
(Byres et al. 2023)	Perspective, Experience	Qualitative, Quantitative	Autism (self)	*n* = 461 Gender (f) = 55% Age: NR	N/A	Public Stigma, Discrimination
(Chen et al. 2013)	Perspective, Experience	Qualitative	Autism (parent)	NR	*n* = 42, Gender (f) = 76% Age: NR	Affiliate Stigma, Discrimination
(Chen et al. 2015)	Perspective	Qualitative	Autism (parent)	NR	*n* = 42, Gender (f) = 76.2% Age: NR	Public Stigma, Self-esteem
(Chen et al. 2020)	Perspective	Quantitative	Autism (parent)	Age: mean (SD) = 9.61(4.88) yrs	*n* = 443, Gender (f) = 75.2% Age: mean = 38.93	Discrimination
(Diedericks et al. 2024)	Perspective	Qualitative	Autism (parent)	NR	*n* = 17 Gender (f) = 12/17 Age: NR	Self Stigma, Labelling
(Fischbach et al. 2016)	Perspective	Quantitative	Autism (parent)	NR	*n* = 502, Gender (f) = 95% Age: mean = 43 yrs, range = 39–47	Public Stigma, Self Stigma
(Fitzgerald et al. 2021)	Experience	Qualitative	RGCs (parent)	NR	*n* = 30 Gender (f) = 80% Age: range = 23–62 yrs	Affiliate Stigma, Isolation
(Gallion et al. 2025)	Perspective, Experience	Qualitative, Quantitative	Autism (self)	*n* = 173 Gender (f): 53.8% Age: range 18–67 yrs	N/A	Public Stigma, Discrimination
(Hayeems et al. 2016)	Experience	Qualitative	Autism (parent)	Age: mean = 5.4 yrs, range = 0–21	*n* = 23 Gender (f) = 82% Age: NR	Self Stigma, Labelling
(Johannessen et al. 2017; Johannessen et al. 2022)	Perspective	Quantitative	Autism (parent)	Gender (f) = 19.5% Age: mean (SD) = 16.5(7.6), range = 3–58	*n* = 1455 Gender (f) = 81% Age: mean = 46.7 yrs, range = 22–87	Public Stigma, Affiliate Stigma, Prejudice, Discrimination
(Klitzman et al. 2024)	Perspective	Qualitative	Autism (self)	*n* = 11 Gender (f): 64% Age: mean (SD) = 29 (7) yrs, range = 22–48	N/A	Public Stigma, Self Stigma,
(Klitzman et al. 2025)	Experience	Qualitative	Autism (parent)	*n* = 22 Gender (f) = 41% Age: mean (SD) = 13.6 (6) yrs, range = 4.5–30.5	*n* = 28 Gender (f) = 53.5% Age: mean (SD) = 47.8 (9) yrs, range = 32–70	Discrimination
(Landlust et al. 2023)	Experience	Quantitative	RGC (PMD) (parent)	NR	*n* = 587	Ignorance
(Lucas et al. 2022)	Perspective, Experience	Quantitative	Autism (parent)	NR	*N* = 211, Gender (f) = 86.4% Age: NR	Isolation
(Li et al. 2016)	Perspective, Experience	Qualitative	Autism (parent)	NR	*n* = 42 Gender (f) = 76% Age: mean = 44 yrs, range = 24–58	Discrimination
(Narcisa et al. 2013)	Perspective, Experience	Quantitative	Autism (parent)	NR	*N* = 162 Gender (f) = 89% Age: range = 25–55	Labelling
(Van Der Steen et al. 2016)	Experience	Qualitative	CNV (parent)	NR	*n* = 12 Gender (f) = 66.7% Age: mean (SD) = 36(4.8) yrs	Public Stigma
(Tremblay et al. 2019)	Perspective, Experience	Qualitative	Autism (parent)	Age range: 0–6 years Age: mean (SD) = 33 m (15 m)	*n* = 57 Gender (f) = 75.5% Age: range = 21–45 yrs	Self Stigma, Isolation
(Trottier et al. 2013)	Experience	Qualitative	Autism (parent)	Age mean = 13	*n* = 9 Gender (f) = 88.9% Age: range = 39–49 yrs	Self Stigma, Ignorance, Prejudice
(Vande Wydeven et al. 2012)	Perspective, Experience	Quantitative, Qualitative	Autism (parent)	Gender (f) = 17.4% Age mean = 9.4	*n* = 155	Public stigma
(Xu et al. 2016)	Perspective	Quantitative, Qualitative	Autism (parent)	Gender (f) = 20.45%	*n* = 45, Gender (f) = 60% Age: mean = 39.1 yrs, range = 24–60	Public Stigma, Ignorance
(Xu et al. 2018)	Perspective	Quantitative	Autism (parent)	NR	*n* = 53, Gender (f) = 73.6% Age: mean = 37.8 yrs, range = 28–56	Discrimination
(Yusuf et al. 2020)	Perspective	Quantitative	Autism (parent)	*n* = 97, Gender (f): 22.7% Age: mean (SD) = 7.09 (3.77) yrs, range = 2.10–17.69.10.69	*n* = 97 Gender (f) = 92.8% Age: mean (SD) = 39 (7.7) yrs	Affiliate Stigma, Public Stigma, Self Stigma

*Perspective relates to participant beliefs about genetic testing; Experience relates participant reported experiences following genetic testing

*CNV* Copy Number Variant, *DD* Developmental Delay, *f *female, *GT* Genetic testing, *m* months, *N*/*A* not applicable, *NR* Not reported, *parent* parent report study, *PMD* Phelan-McDermid Syndrome; *RGC* Rare Genetic Condition, *self* self-report study, *yrs* Years

In a qualitative study examining the experiences of twelve parents of children with autism-related CNV identified during prenatal testing, many parents did not report stigma or persistent concerns about their child’s development following genetic testing results, noting their worries were no different to typical parent concerns, while one parent reported stigmatising their child due to their genetic variant when they displayed unusual behaviours, despite not having a clinical diagnosis (Van Der Steen et al. 2016).

Attitudes of parents with at least one autistic child (*n* = 42) towards prenatal genetic testing indicated mixed findings, particularly regarding hypothetical decisions about family planning and pregnancy (Chen et al. 2015). While over half of the parents believed that a prenatal test would be useful in preparing for their child’s birth, five stated that a positive genetic test could influence their pregnancy decisions due to societal stigma and the anticipated burden on the child, family, and society.

### Self-Stigma

#### Autistic people’s perspectives

Autistic participants reported the belief that genetic testing would reduce self-stigma and blame if autism was viewed as genetically determined and not entirely within the individual’s control. They felt that this could positively affect masking behaviours, which often occur as a consequence of stigma and negative attitudes towards autism (Klitzman et al. 2024).

#### Parental perspectives and experiences

Although parental self-stigma was not specifically referred to in any study, many parents described internalised blame or guilt about their parenting, and expressed mixed findings regarding their perceived influence of a genetic test result in six studies. In one study, 24% of parents reported that a genetic finding would make them feel more responsible for their child’s autism (Fischbach et al. 2016). Yusuf et al. (2020) reported that 45% of parents agreed and 25% strongly agreed that they would feel guilty if they passed on a biological risk to their child. In a study of 19 parents, some parents also indicated that self-blame or guilt would be exacerbated particularly with inheritance, although many parents expressed how a genetic test result could reduce their self-blame (Diedericks et al. 2024). Similarly, in a study of 57 parents of children with a developmental condition, 11% of parents reported feelings guilt associated with transmission, while 11% also reported that it would reduce parental guilt and blame (Tremblay et al. 2019). In a study of 23 parents, relief with not being to blame was also mentioned (Hayeems et al. 2016).

### Affiliate stigma

Three articles described the experience of affiliate stigma by parents. Most parents (86%) in the study by Yusuf et al. (2020) either agreed or strongly agreed that the results of genetic testing can stigmatise families. In a separate study (*n* = 42), eleven parents were against genetic testing in autism, with four of those indicating that overemphasizing the genetic link could have a negative impact on stigma for families (Chen et al. 2013). Affiliate stigma was also reported in a study of thirty parents of children with autism-related genetic conditions (Fitzgerald et al. 2021).

### Stigma-related factors

Stigma-related factors were reported in two autistic self-report studies and fourteen parent report studies. Labelling, discrimination, prejudice, isolation, ignorance, and self-esteem were discussed in relation to genetic testing in autism and related genetic conditions, however, other commonly described stigma related factors such as stereotyping, marginalisation, rejection or status loss did not appear in the results of any of the studies included as part of this scoping review (Table 2).

#### Discrimination & prejudice

Autistic participants in two studies believed that genetic testing would increase discrimination towards autistic people, with the majority (83%) of participants in one study agreeing (Byres et al. 2023), and more than half in another study (57.8%) also agreeing that genetic testing could lead to discrimination (Gallion et al. 2025).

Discrimination and prejudice were reported in eight parent-report studies, although the proportion of parents indicating negative impacts of genetic testing differed across studies. In one study, a few parents (4/42), who were against genetic testing for their child, reported concerns that positive results could lead to discrimination in health or life insurance settings (Chen et al. 2013). In another study of forty-two parents, 12% of participants also raised concerns about discrimination, including the potential for future exclusion of their child from insurance coverage (Li et al. 2016). In a large Norwegian cohort study (*n* = 1455), 56–67% of parent participants also expressed concerns about insurance discrimination due to genetic testing, even though such discrimination is legally prohibited in healthcare (Johannessen et al. 2017; Johannessen et al. 2022). In contrast, some parents in another indicated the hope that social service agencies and insurers might view their child’s condition as more medical than behavioral, potentially justifying greater coverage and supports (Klitzman et al. 2025).

In a study assessing the impact of web-based educational modules on parental understanding of clinical genetic testing, the proportion of parents who believed discrimination could result from sharing test results increased from 19% to 28% after completing the module (Xu et al. 2018).

In another study of attitudes towards prenatal genetic testing (*n* = 443), many parents indicated genetic testing would lead to social discrimination, however the context of the discrimination was not explored further (Chen et al. 2020).

Finally, parents of non-speaking autistic people (*n* = 20), reported a positive impact of genetics, such that a genetic explanation could have helped them mitigate judgment from others (Asbury et al. 2024).

#### Isolation

Isolation was reported in two studies; 13% parents of children with a developmental condition reported isolation related to lack of knowledge about who can support them and how to access services without a genetic diagnosis (Tremblay et al. 2019), while some parents of children with rare genetic conditions reported feeling unseen and isolated in social and healthcare settings, with feelings of isolation reduced when they connected with other parents of children with the same genetic diagnosis (Fitzgerald et al. 2021).

#### Ignorance

Four parent studies mentioned ignorance. In the context of healthcare professionals, a large sample study (*n* = 597) focused on Phelan McDermid Syndrome, about a third (37%) of parents reported encountering a clinician who lacked knowledge about their child’s genetic condition as extremely stressful (Landlust et al. 2023). As previously mentioned, three studies mentioned their hope that genetic testing research would reduce public ignorance towards autism (Xu et al. 2016; Xu et al. 2018; Trottier et al. 2013).

#### Labelling

In a study of 162 parents, 12% of parents of at least one autistic child who were not interested in genetic testing for their undiagnosed younger children reported concern that a genetic test result would cause their child to be labelled (Narcisa et al. 2013). However, labelling was also identified as a positive in two studies. In a smaller study, it was perceived that genetic testing could lead to a definitive diagnosis could guide treatment and enable parents to feel more empowered, rather than negative stigma-related impacts attributed to labelling, as they could learn about the condition (Diedericks et al. 2024). Finally, an experience reported by one parent in another study, stated that their child would not have access to their current healthcare services without their genetic diagnosis (Hayeems et al. 2016).

##### Self-esteem

In the context of prenatal testing, parents reported that parental self-esteem may be impacted by having an autistic child due to societal and cultural attitudes towards neurodevelopmental conditions (Chen et al. 2015).

### Mental health & Well-being

As a secondary aim, we explored whether the impact of genetic testing on stigma subsequently affected mental health and well-being. Autistic adults in one study indicated that a genetic test result could provide a biological meaning to autism and subsequently alleviate some of the stress associated with autism related stigma and any feelings of internalised blame (Klitzman et al. 2024).

Parent findings were mixed. Some parents expressed anxiety about the possibility of inheritance of a condition (Tremblay et al. 2019; Diedericks et al. 2024), however, as mentioned previously Diedericks et al. (2024) reported that most parents felt a genetic diagnosis could relieve feelings of blame or guilt regarding their child’s clinical outcomes.

Parents of people with Phelan McDermid Syndrome disclosed that genetic testing could lead to increased concern and stress related to potential insurance challenges, suggesting feelings that genetic testing-related discrimination could exacerbate psychological stress (Landlust et al. 2023). Parents of people with rare genetic diagnosis in another study discussed how feeling isolated and lack of clinician follow-up impacted on mental health, along with influences of poor access to information about outcomes on parental fears and expectations for their child’s future outcomes (Fitzgerald et al. 2021).

A direct impact of genetic testing on stigma and subsequent mental health was not reported in some studies. For example, while half of the parents in one study reported their experience of feeling shocked initially following genetic testing results, the majority reported they would still choose to undergo genetic testing and receive results again (Van Der Steen et al. 2016).

Although not directly related to stigma, numerous studies highlighted an increase in parental stress and anxiety related to genetic testing, including the impact of the test result, and receiving a diagnosis (Tremblay et al. 2019; Lucas et al. 2022). In the context of prenatal testing to test for genetic conditions associated with increased risk for autism, some parents reported that it could increase stress during pregnancy (Chen et al. 2015), while in other studies parents suggested that the genetic testing result could provide relief and reduce stress in preparation for their child’s birth (Xu et al. 2016; Xu et al. 2018), or would enhance their ability to cope with their child’s autism diagnosis and improve their sense of empowerment as caregivers (Lucas et al. 2022).

## Discussion

### Overview of findings

The aim of this scoping review was to identify the extent to which existing research has explored the impact of genetic testing on stigma and stigma-related factors in autism and autism-related conditions. The overall findings indicate that genetic testing influences both attitudes toward, and experiences of autism-related stigma for both autistic people and parents of autistic people. Although few studies have addressed autistic adult perspectives, the divergence in autistic people’s perspectives both within and between studies, and compared to parental studies, suggests there is wide variability across autistic people and their parents or caregivers in relation to attitudes towards genetic testing and the impact on stigma in autism. Key areas that emerged in relation to stigma and genetic testing were positive and negative influences on family and societal acceptance and awareness, as well as concerns about increased discrimination. Discrimination issues were also expressed by a subset of autistic adults (17%) in a recent study published following our literature search (Hendry et al. 2025). Given this study and the included autistic adult studies were all published within the past two years, these concerns remain highly relevant to research, clinical practice and policy. The findings of negative, positive, and neutral impacts of genetic testing on self- and public- stigma in autism suggest that all autistic people and their parents or caregivers may not necessarily have a single unified perspective towards genetic testing. Although limited in number, findings across studies suggest some important distinctions between autistic people’s and parent perceptions, which are crucial for understanding the psychosocial impacts of genetic testing on family members.

Mixed parental perspectives included beliefs that genetic testing can help to enhance biological understanding of autism and increase acceptance and awareness, while others raised worries about societal discrimination, labelling, and stigma despite not meeting the clinical criteria for a diagnosis of autism (Johannessen et al. 2017). Some parents described concerns about insurance-related discrimination, which has been identified as a decisional barrier, leading to appeals for legislative action on genetic testing and health insurance (Rothenberg 1997). These perspectives, along with parental experiences of isolation, and self-blame related to inheritance suggests that careful consideration of clinical care and genetic counselling for families is necessary to balance the benefits of genetic testing and to prevent any increase in stigma and related factors (Fitzgerald et al. 2021, Zhong et al. 2021). The findings across studies highlight the importance of respecting autonomy and addressing any community concerns (Soini 2012; Morris et al. 2022).

### Gaps in knowledge

Several literature gaps were identified. While three studies included autistic adults, our literature search did not identify any studies examining perspectives or experiences of autistic adolescents. Research needs to further explore attitudes of autistic adults and adolescents, and whether they may differ across cultures or countries, to better understand feelings about the impacts of research and clinical genetic testing on public and self-stigma. Longitudinal studies could also examine whether youth maintain the same perspective as they transition into adulthood. In addition, there is a need for inclusion of non-speaking autistic people’s perspectives in genomic research and genetic testing, as their challenges are often different to autistic adults who can advocate for themselves (Asbury et al. 2024). Parents of non-speaking autistic people have described feelings of isolation and perceived marginalisation within the broader autism community, particularly relating the underrepresentation of non-speaking autistic people’s (and their parents) perspectives (Asbury et al. 2024). The challenges experienced and care supports for autistic people can also be highly individualised, which may further influence attitudes towards genetic testing and whether it exacerbates or relieves stigma. Further research is necessary to gather the diverse perspectives and experiences across autistic youth, adults, both speaking and minimally or non-speaking, to adequately represent the range of attitudes towards stigma impacts of genetic testing in autism. It will also be important to compare the perspectives of autistic people and their parents, including ethical considerations regarding who makes the decision and when to pursue testing (Hendry et al. 2025).

A small proportion of autistic individuals in two studies included in the scoping review had undergone genetic testing, highlighting another key under-represented population. One study reported on feelings towards genetic testing impacts between those who had and had not received a genetic test (Byres et al. 2023). Research on psychosocial factors, including the experience of stigma among individuals diagnosed with rare genetic conditions or carrying autism-related rare variants, is essential to determine whether their genetic diagnosis reinforces or alleviates self-stigma and influences their perceptions of public stigma. It would also be useful to explore whether genetic testing may reinforce negative perceptions in cases where clinical criteria for an autism diagnosis are not met, as indicated by one parental study (Johannessen et al. 2017), and to also compare to those who do meet clinical criteria for autism diagnosis and do not receive a positive genetic result following testing. Moreover, comparisons between rare genetic diagnoses are lacking.

As highlighted in the studies reported in this scoping review, a genetic result that can be perceived to explain an autism diagnosis can be associated with feelings of blame or guilt. These feelings can contribute to stigma, such that parents may either blame themselves or believe others will blame them for their child’s condition, and consequently also worry about social rejection (Zhou et al. 2018; Burrell et al. 2017; Diedericks et al. 2024). This is also very relevant in the context of variants that are inherited or carried by multiple family members (Johannessen et al. 2017). An additional complexity is that many variants associated with autism are associated with heterogeneous outcomes with varying levels of impact on quality of life. To date no studies have investigated how stigma is experienced in the context of inherited variants with variable outcomes (Merikangas et al. 2015; Belyaeva and Lebedev 2022). Similarly, investigating how carriers of autism-related CNVs with varying clinical phenotypes within families (including both affected and unaffected parent and sibling carriers) experience stigma is crucial to understanding differences in the psychosocial impacts of their genetic diagnosis. This may inform counselling needs for families with complex clinical profiles, and may help to understand and alleviate any self, public or affiliate-stigma experienced by family members.

Several hundred rare variants are associated with autism and other neurodevelopmental conditions, which is enhancing our understanding of the underlying mechanisms that may be altered (Antaki et al. 2022; Rolland et al. 2023; Wilfert et al. 2021). However, the helplessness felt by parents reflects a lack of adequate training of healthcare professionals in this fast-moving field. Isolation and ignorance among clinicians are key considerations for parents of people with rare genetic conditions (Fitzgerald et al. 2021; Landlust et al. 2023). Poor clinician training and communication regarding genetic diagnoses emphasises the need to improve knowledge in this area. Gaps in access to genetic counselling leave parents feeling lost and alone (Fitzgerald et al. 2021). The parental experiences highlighted the need for enhanced training and education for healthcare professionals to better equip them with necessary skills to support patients receiving a clinical genetic diagnosis, as well as access the timely to genetic counselling services. The need for enhanced training in genetic testing practices has also been separately reported by healthcare professionals (Wang et al. 2024).

Beyond diagnostic utility, genetic testing appears to have some psychosocial benefits for some autistic adults by providing a biological explanation that reduces internalised stigma and self-blame (Klitzman et al. 2024). This suggests the importance of ethical and sensitive communication, with consideration of potential psychosocial impacts of autism-related genetic results. Mental health outcomes for parents and their children were mixed, and the contributory factors were complex. One significant factor identified was the cultural and societal impact of stigma on family planning, and the increased stress and worry following prenatal genetic testing. Feelings of guilt or blame, personal beliefs, familial dynamics, and broader social contexts may all impact parental responses and well-being following clinical genetic testing. Clearly, appropriate clinical support and education is crucial pre-and post-genetic testing to reduce any stigma impacts (Dimillo et al. 2013).

Genetic counselling has been shown to provide meaningful impact on the psychosocial aspects of genetic testing, such as promoting a sense of empowerment (Austin et al. 2014; Edwards et al. 2008). Resources explaining genetic testing and psychoeducational materials for rare neurodevelopmental-associated genetic conditions may also help reduce negative impacts on mental health and well-being and increase societal acceptance of autistic people with rare genetic variants. Organisations such Unique (https://rarechromo.org/) and Simon Foundation’s SPARK (https://sparkforautism.org) have developed many resources for families to access and understand their child’s genetic condition and the variability in prognosis. In addition to parent resources, tools are also needed for autistic people and people with autism-related genetic conditions, to help them to understand and manage their own experiences of stigma and feelings surrounding genetic testing, as well as subsequent mental health outcomes (Summers et al. 2024). This aligns with a recent report from the Embracing Complexity Network on top research priorities for neurodivergent people to understand their own needs (https://embracingcomplexity.org.uk/reports/top-10-research-priorities).

### Limitations

Several limitations were identified. First, the majority of included articles reflected parental perspectives, with autistic adults’ views examined in three recent articles only. The more recent articles focused on autistic adult perspectives may suggest a shift in autism research focus, toward participatory and neurodiversity-affirming approaches. Secondly, the current review was limited to articles published in English, which may have resulted in exclusion of relevant articles from non-English speaking countries. Finally, most of the studies represent research conducted in North America, which may not capture the experiences, social or cultural contexts of genetic testing in autism for diverse populations worldwide.

### Future directions

Sensitive topics such as genetic testing should be discussed through meaningful consultation with representatives of the relevant communities (i.e. autistic people, and parents of autistic people) to ensure that research aligns with their priorities. Autism community engagement should include the diverse views from autistic people and their family members who may also be affected by a genetic test result (Asbury et al. 2024), with careful attention paid to situations where priorities differ between different relevant communities. Recently, the Spectrum 10 K study received significant criticism for lacking clear aims to improve the well-being of autistic people and for failing to incorporate consultation with the autism community to define the study research goals (https://spectrum10k.org/about-spectrum-10k/). Although a consultation process with over 500 autistic individuals and caregivers was subsequently introduced, the intended study was ultimately closed. Instead, the research team announced plans to use existing genetic and health data to research mental and physical health priorities identified through consultation. This has resulted in a clear lesson for the research community, especially in the context of genetic testing, to ensure collaboration with community representatives at all stages of research, from co-design of the study to the interpretation and dissemination of findings, to effectively translate the benefit to clinical care or well-being of autistic individuals (Tindall et al. 2021; O’Brien et al. 2021).

Research consortia have made considerable progress through consultation with autism and rare genetic community representatives to advocate for neuro-affirmative biomedical autism research, and efforts to understand and focus on community research priorities for therapeutic interventions and genomics informed clinical trials (Bloomfield et al. 2023; Heraty et al. 2023). There have also been efforts to understand autistic people’s perspectives, along with carers and professional’s perspectives, to identify barriers within autism care pathways and understand how to improve healthcare experiences (Mendez et al. 2023). Future research and care pathways must be guided by the priorities and needs of the community in a way that does not contribute to stigma. In addition, the perspectives of autistic communities worldwide need to be gathered, including non-English speaking countries, to ensure that cultural contexts and nuances are captured.

The current review findings highlight important clinical implications, particularly for interactions with families about genetic testing in the context of autism. The variability in perceptions across family members, underscores the importance of navigating the influence of stigma and discrimination on the views of genetic testing within clinical interactions on this topic. Incorporation of approaches used by genetic counsellors that are sensitive to stigma, from pre-genetic test to post-genetic test stages of the process, could help to ensure that information, communication and supports address individual and family needs. This includes discussing beliefs and motivations, and addressing concerns around genetic testing or potential differing opinions, to tailor counselling sessions to support the autistic individual and their family (Gallion et al. 2025). Psychosocial supports greatly help families to process the expectations and the outcomes of a clinical genetic test (both genetic or no genetic result) and any potential feelings of blame, discrimination or stigma. Embedding these genetic counselling approaches into all clinical care would enhance person-centred care.

## Conclusion

This scoping review identified a need for further research exploring the attitudes of autistic people broadly, including youth, and among people with autism-associated genetic conditions and their families to inform clinical care and genomics research. Genetic testing in the context of autism and related conditions needs to be considered in the context of neuro-affirmative care (Leblond et al. 2024). Co-designing research and clinical care with neurodivergent people can help to identify the context in which a genetic diagnosis is of value and how it aligns with their healthcare needs (Tindall et al. 2021). Along with enhancements in clinical care, it is crucial for research to also focus on better societal acceptance, and to implement strategies to reduce stigma around autism and genetic testing.

## Supplementary Information

Below is the link to the electronic supplementary material.

Supplementary Material 1

## Data Availability

No data were generated or analysed as part of this scoping review.
