# Supplementary material for: Does genetic testing impact stigma in autism: A scoping review
Source: J Community Genet. 2026 Jan 28;17(1):25. doi: 10.1007/s12687-025-00854-8 (PMC12852527; doi:10.1007/s12687-025-00854-8)
Supplement: Supplementary file 1 — Supplementary Material 1 [file 12687_2025_854_MOESM1_ESM.docx]

Supplementary Methods: Literature Search Strategy

**Inclusion Criteria for Autism-related Genetic Variants and Syndromes**

| Table 1. Autism-related copy number variant and rare genetic syndrome list for article inclusion based on research literature (Coe et al., 2014, Yoo, 2015, Kendall et al., 2019). | |
| --- | --- |
| 1p36 del. | 16p11.2 dup. |
| 1p36 dup. | 17p13.3 del. (*YWHAE*) |
| 1q21.1 del. | 17p13.3 dup. (*YWHAE*) |
| 1q21.1 dup. | 17p13.3 del. (*PAFAH1B1*) |
| 2p16.3 del. (NRXN1) | 17p13.3 dup. (*PAFAH1B1*) |
| 2q21.2 del. | 17q11.2 del. (Smith-Magenis Syndrome) |
| 2q37 del. | 17p11.2 dup. (Potocki-Lupski) |
| 3q29 del. | 17q11.2 del (*NF1*) |
| 4p16.3 del. (Wolf-Hirschhorn) | 17q11.2 dup. (*NF1*) |
| 4p16.3 dup. (Wolf-Hirschhorn) | 17q12 dup. |
| 5q35 del. (Sotos syndrome) | 17q12 del. |
| 7q11.23 del. (William-Beuren syndrome) | 17q21.31 del. |
| 7q11.23 dup. (William-Beuren syndrome) | 22q11.2 del. |
| 8p23.1 del. | 22q11.2 dup. |
| 8p23.1 dup. | 22q11.2 distal del. |
| 9q34.3 del. (EHMT1) | 22q11.2 distal dup. |
| 10q23 del. | 22q13 del.( *SHANK3*) |
| 11p11.2 del. (Potocki-Shaffer) | 22q13 dup. (*SHANK3*) |
| 15q11.2 del. | 23q13.3 del. *(Phelan-Mcdermid syndrome)* |
| 15q11-q13 del. (Prader-Willi/Angelman Syndrome) | FMR1 *(fragile X syndrome)* |
| 15q11-q13 dup. (Prader-Willi/Angelman syndrome) | MECP2 *(Rett’s syndrome)* |
| 15q13.3 del. BP4-BP5 | TSC1, TSC2 (Tuberous sclerosis) |
| 15q24 del. | NF1 (*Neurofibromatosis*) |
| 15q24 dup. | SMCIA/SMC3 (*Cornelia de Lange syndrome*) |
| 15q25 del. | COH1 (*Cohen syndrome*) |
| 16q13.11 del. | DHCR7 (*Smith Lemli-Opitz syndrome*) |
| 16q13.11 dup. | *Down Syndrome* |
| 16p12.1 del. | CHD7 *(CHARGE Syndrome)* |
| 16p11.2 distal del. | *Duchenne Muscular Dystrophy* |
| 16p11.2 distal dup. | NSD1 *(Sotos Syndrome)* |
| 16p11.2 del. |  |

**Sickkids Library Initial Search Strategy**

Table 2. Sickkids Library Literature Search Strategy

| **Date** | May 24, 2023 |
| --- | --- |
| **Topic** | Impact of genetic testing on stigma in autism |
| **Date limit(s)** | n/a |
| **Language** | English |
| **Other limits** | Omit Conference abstracts |

**Sickkids Library Initial Search Results**

Table 3. Summary of articles retrieved from databases.

| **Database [Platform]** *Search run May 30, 2023* | **Results** |
| --- | --- |
| Ovid APA PsycInfo <1806 to May Week 4 2023> | 196 |
| Ovid MEDLINE(R) and Epub Ahead of Print, In-Process, In-Data-Review & Other Non-Indexed Citations and Daily <1946 to May 25, 2023> | 209 |
| Ovid Embase Classic+Embase <1947 to 2023 Week 20> | 341 |
| **TOTAL (duplicates not removed)** | **746** |

Table 4. APA PsycInfo <1806 to May Week 4 2023>.

| **#** | **Searches** | **Results** |
| --- | --- | --- |
| 1 | exp Autism Spectrum Disorders/ or ((Autis* or ASD or Asperger*) adj3 (disease* or disorder* or syndrom*)).mp. | 62271 |
| 2 | Epigenetics/ or Genetics/ or Genetic Testing/ or "Genetic Counseling"/ | 53618 |
| 3 | ("genetic test*" or "Chromosomal Microarray Analy*" or CMA or "genetic/epigenetic" or epigenesis* or epigenetic* or "genetic screen*" or "genetic predispos*" or "Genetic Predictive Test*" or "Genetic Counsel*" or "heredity counsel*" or "genetic guidance*").tw,hw. | 17982 |
| 4 | 2 or 3 | 61713 |
| 5 | exp Stigma/ or *Attitudes/ or "Health Attitudes"/ or exp "Stereotyped Attitudes"/ or exp Discrimination/ or exp Prejudice/ | 124900 |
| 6 | (stigma* or stereotyp* or "self-stigma*" or discriminat* or ableis* or prejudic* or bias* or "social judgement*").tw,hw. | 344311 |
| 7 | "Coping behavior"/ or Adjustment/ or "Emotional Adjustment"/ or "Self-Worth"/ or "Health Knowledge"/ or (coping or cope$ or "self-worth" or "lived experience*" or "Parent* perspective*" or fear* or "risk/pain" or pain).tw,hw. | 377716 |
| 8 | ((mental* or emotional* or psychological*) adj2 (adapt* or health* or experience* or perspective* or attitude*)).tw,hw. | 311323 |
| 9 | or/5-8 | 974671 |
| 10 | 1 and 4 and 9 | 288 |
| 11 | limit 10 to ("0200 book" or "0240 authored book" or "0280 edited book" or "0300 encyclopedia" or "0400 dissertation abstract") | 79 |
| 12 | limit 10 to conference proceedings | 1 |
| 13 | 11 or 12 | 79 |
| 14 | 10 not 13 | 209 |
| 15 | limit 14 to english language | 196 |
| 16 | remove duplicates from 15 | 196 |

Table 5. Ovid MEDLINE(R) and Epub Ahead of Print, In-Process, In-Data-Review & Other Non-Indexed Citations and Daily <1946 to May 25, 2023>

| **#** | **Searches** | **Results** |
| --- | --- | --- |
| 1 | exp Autism Spectrum Disorder/ or ((Autis* or ASD or Asperger*) adj3 (disease* or disorder* or syndrom*)).mp. | 61100 |
| 2 | Epigenesis, Genetic/ or *Genetics/ or Genetic Testing/ or "Genetic Counseling"/ or *epigenomics/ | 113251 |
| 3 | ("genetic test*" or "Chromosomal Microarray Analy*" or CMA or "genetic/epigenetic" or epigenesis* or epigenetic* or "genetic screen*" or "genetic predispos*" or "Genetic Predictive Test*" or "Genetic Counsel*" or "heredity counsel*" or "genetic guidance*" or "genetic educat*").tw,kw,kf. | 200887 |
| 4 | 2 or 3 | 250209 |
| 5 | exp "Social Stigma"/ or "Health Knowledge, Attitudes, Practice"/ or *Attitude/ or exp "Stereotyping"/ or exp "Social Discrimination"/ or exp Prejudice/ | 203786 |
| 6 | (stigma* or stereotyp* or "self-stigma*" or discriminat* or ableis* or prejudic* or bias* or "social judgement*").tw,kw,kf. | 652365 |
| 7 | "Adaptation, psychological"/ or "Emotional Adjustment"/ or "Self concept"/ or (coping or cope$ or "self-worth" or "lived experience*" or "Parent* perspective*" or fear* or "risk/pain" or pain).tw,kw,kf. | 1104540 |
| 8 | ((mental* or emotional* or psychological*) adj2 (adapt* or health* or experience* or perspective* or attitude*)).tw,kw,kf. | 259580 |
| 9 | or/5-8 | 2065627 |
| 10 | 1 and 4 and 9 | 237 |
| 11 | limit 10 to english language | 220 |
| 12 | (conference abstract or "Case Reports" or editorial or comment or letter or newspaper article).pt. | 4288683 |
| 13 | 11 not 12 | 209 |
| 14 | remove duplicates from 13 | 209 |

Table 6. Embase Classic+Embase <1947 to 2023 Week 20>

| **#** | **Searches** | **Results** |
| --- | --- | --- |
| 1 | exp Autism/ or ((Autis* or ASD or Asperger*) adj3 (disease* or disorder* or syndrom*)).mp. | 103194 |
| 2 | genetic epigenesis/ or *Genetics/ or Genetic Screening/ or "Genetic Counseling"/ or *epigenetics/ | 309773 |
| 3 | ("genetic test*" or "Chromosomal Microarray Analy*" or CMA or "genetic/epigenetic" or epigenesis* or epigenetic* or "genetic screen*" or "genetic predispos*" or "Genetic Predictive Test*" or "Genetic Counsel*" or "heredity counsel*" or "genetic guidance*" or "genetic educat*").tw,kw,kf. | 299105 |
| 4 | or/2-3 | 492077 |
| 5 | exp "Social Stigma"/ or "attitude to health"/ or *Attitude/ or exp "Stereotyping"/ or exp "Social Discrimination"/ or exp Prejudice/ | 210135 |
| 6 | (stigma* or stereotyp* or "self-stigma*" or discriminat* or ableis* or prejudic* or bias* or "social judgement*").tw,kw,kf. | 826820 |
| 7 | "psychological adjustment"/ or "Self concept"/ or (coping or cope$ or "self-worth" or "lived experience*" or "Parent* perspective*" or fear* or "risk/pain" or pain).tw,kw,kf. | 1593815 |
| 8 | ((mental* or emotional* or psychological*) adj2 (adapt* or health* or experience* or perspective* or attitude*)).tw,kw,kf. | 322441 |
| 9 | or/5-8 | 2770045 |
| 10 | 1 and 4 and 9 | 531 |
| 11 | limit 10 to english language | 502 |
| 12 | limit 11 to (books or chapter or conference abstract or conference paper or "conference review" or editorial or erratum or letter or note or short survey or tombstone) | 146 |
| 13 | 11 not 12 | 356 |
| 14 | remove duplicates from 13 | 341 |

**Sickkids Library Updated Search Results**

Table 7. Summary of articles retrieved from databases.

| **Database [Platform]** *Search updated January 9, 2025* | **Results** |
| --- | --- |
| Ovid APA PsycInfo <1806 to December 2024 Week 5> | 16 |
| Ovid MEDLINE(R) and Epub Ahead of Print, In-Process, In-Data-Review & Other Non-Indexed Citations and Daily <1946 to January 6, 2025> | 40 |
| Ovid Embase Classic+Embase <1947 to 2025 Week 01> | 85 |
| **TOTAL (duplicates not removed)** | **141** |

Table 8. Ovid APA PsycInfo <1806 to December 2024 Week 5>

| **#** | **Searches** | **Results** |
| --- | --- | --- |
| 1 | exp Autism Spectrum Disorders/ or ((Autis* or ASD or Asperger*) adj3 (disease* or disorder* or syndrom*)).mp. | 69234 |
| 2 | Epigenetics/ or Genetics/ or Genetic Testing/ or "Genetic Counseling"/ | 56584 |
| 3 | ("genetic test*" or "Chromosomal Microarray Analy*" or CMA or "genetic/epigenetic" or epigenesis* or epigenetic* or "genetic screen*" or "genetic predispos*" or "Genetic Predictive Test*" or "Genetic Counsel*" or "heredity counsel*" or "genetic guidance*").tw,hw. | 19584 |
| 4 | 2 or 3 | 65103 |
| 5 | exp Stigma/ or *Attitudes/ or "Health Attitudes"/ or exp "Stereotyped Attitudes"/ or exp Discrimination/ or exp Prejudice/ | 135750 |
| 6 | (stigma* or stereotyp* or "self-stigma*" or discriminat* or ableis* or prejudic* or bias* or "social judgement*").tw,hw. | 373833 |
| 7 | "Coping behavior"/ or Adjustment/ or "Emotional Adjustment"/ or "Self-Worth"/ or "Health Knowledge"/ or (coping or cope$ or "self-worth" or "lived experience*" or "Parent* perspective*" or fear* or "risk/pain" or pain).tw,hw. | 409829 |
| 8 | ((mental* or emotional* or psychological*) adj2 (adapt* or health* or experience* or perspective* or attitude*)).tw,hw. | 351917 |
| 9 | or/5-8 | 1063569 |
| 10 | 1 and 4 and 9 | 314 |
| 11 | limit 10 to ("0200 book" or "0240 authored book" or "0280 edited book" or "0300 encyclopedia" or "0400 dissertation abstract") | 81 |
| 12 | limit 10 to conference proceedings | 1 |
| 13 | 11 or 12 | 81 |
| 14 | 10 not 13 | 233 |
| 15 | limit 14 to english language | 215 |
| 16 | limit 15 to yr="2023 - 2025" | 16 |
| 17 | remove duplicates from 16 | 16 |

Table 9. Ovid MEDLINE(R) Epub Ahead of Print and In-Process, In-Data-Review & Other Non-Indexed Citations and Daily 1946-January 06, 2025

| **#** | **Searches** | **Results** |
| --- | --- | --- |
| 1 | exp Autism Spectrum Disorder/ or ((Autis* or ASD or Asperger*) adj3 (disease* or disorder* or syndrom*)).mp. | 69636 |
| 2 | Epigenesis, Genetic/ or *Genetics/ or Genetic Testing/ or "Genetic Counseling"/ or *epigenomics/ | 121928 |
| 3 | ("genetic test*" or "Chromosomal Microarray Analy*" or CMA or "genetic/epigenetic" or epigenesis* or epigenetic* or "genetic screen*" or "genetic predispos*" or "Genetic Predictive Test*" or "Genetic Counsel*" or "heredity counsel*" or "genetic guidance*" or "genetic educat*").tw,kw,kf. | 231285 |
| 4 | 2 or 3 | 281513 |
| 5 | exp "Social Stigma"/ or "Health Knowledge, Attitudes, Practice"/ or *Attitude/ or exp "Stereotyping"/ or exp "Social Discrimination"/ or exp Prejudice/ | 216927 |
| 6 | (stigma* or stereotyp* or "self-stigma*" or discriminat* or ableis* or prejudic* or bias* or "social judgement*").tw,kw,kf. | 740978 |
| 7 | "Adaptation, psychological"/ or "Emotional Adjustment"/ or "Self concept"/ or (coping or cope$ or "self-worth" or "lived experience*" or "Parent* perspective*" or fear* or "risk/pain" or pain).tw,kw,kf. | 1221411 |
| 8 | ((mental* or emotional* or psychological*) adj2 (adapt* or health* or experience* or perspective* or attitude*)).tw,kw,kf. | 309069 |
| 9 | or/5-8 | 2309257 |
| 10 | 1 and 4 and 9 | 281 |
| 11 | limit 10 to english language | 263 |
| 12 | (conference abstract or "Case Reports" or editorial or comment or letter or newspaper article).pt. | 4542960 |
| 13 | 11 not 12 | 249 |
| 14 | remove duplicates from 13 | 248 |
| 15 | limit 14 to dt=20230525-20250109 | 38 |
| 16 | limit 14 to ed=20230525-20250109 | 21 |
| 17 | limit 14 to ez=20230525-20250109 | 37 |
| 18 | or/15-17 | 40 |

Table 10. Ovid Embase Classic+Embase <1947 to 2025 Week 01>

| **#** | **Searches** | **Results** |
| --- | --- | --- |
| 1 | exp Autism/ or ((Autis* or ASD or Asperger*) adj3 (disease* or disorder* or syndrom*)).mp. | 117276 |
| 2 | genetic epigenesis/ or *Genetics/ or Genetic Screening/ or "Genetic Counseling"/ or *epigenetics/ | 335096 |
| 3 | ("genetic test*" or "Chromosomal Microarray Analy*" or CMA or "genetic/epigenetic" or epigenesis* or epigenetic* or "genetic screen*" or "genetic predispos*" or "Genetic Predictive Test*" or "Genetic Counsel*" or "heredity counsel*" or "genetic guidance*" or "genetic educat*").tw,kw,kf. | 336656 |
| 4 | 2 or 3 | 541244 |
| 5 | exp "Social Stigma"/ or "attitude to health"/ or *Attitude/ or exp "Stereotyping"/ or exp "Social Discrimination"/ or exp Prejudice/ | 234415 |
| 6 | (stigma* or stereotyp* or "self-stigma*" or discriminat* or ableis* or prejudic* or bias* or "social judgement*").tw,kw,kf. | 919602 |
| 7 | "psychological adjustment"/ or "Self concept"/ or (coping or cope$ or "self-worth" or "lived experience*" or "Parent* perspective*" or fear* or "risk/pain" or pain).tw,kw,kf. | 1751829 |
| 8 | ((mental* or emotional* or psychological*) adj2 (adapt* or health* or experience* or perspective* or attitude*)).tw,kw,kf. | 377277 |
| 9 | or/5-8 | 3066640 |
| 10 | 1 and 4 and 9 | 643 |
| 11 | limit 10 to english language | 613 |
| 12 | limit 11 to (books or chapter or conference abstract or conference paper or "conference review" or editorial or erratum or letter or note or short survey or tombstone) | 183 |
| 13 | 11 not 12 | 430 |
| 14 | remove duplicates from 13 | 420 |
| 15 | limit 14 to dc=20230525-20250109 | 85 |

**References:**

Coe, B. P., Witherspoon, K., Rosenfeld, J. A., Van Bon, B. W., Vulto-Van Silfhout, A. T., Bosco, P., et al. 2014. Refining analyses of copy number variation identifies specific genes associated with developmental delay. *Nat Genet,* 46**,** 1063-71, doi:10.1038/ng.3092.

Kendall, K. M., Rees, E., Bracher-Smith, M., Legge, S., Riglin, L., Zammit, S., et al. 2019. Association of Rare Copy Number Variants With Risk of Depression. *JAMA Psychiatry,* 76**,** 818-825, doi:10.1001/jamapsychiatry.2019.0566.

Yoo, H. 2015. Genetics of Autism Spectrum Disorder: Current Status and Possible Clinical Applications. *Exp Neurobiol,* 24**,** 257-72, doi:10.5607/en.2015.24.4.257.
